# Gene expression signature for early prediction of late occurring pancytopenia in irradiated baboons

**DOI:** 10.1007/s00277-017-2952-7

**Published:** 2017-02-24

**Authors:** Matthias Port, Francis Hérodin, Marco Valente, Michel Drouet, Andreas Lamkowski, Matthäus Majewski, Michael Abend

**Affiliations:** 1 Bundeswehr Institute of Radiobiology affiliated to the University of Ulm, Neuherbergstr. 11, 80937 Munich, Germany; 2 Institut de Recherche Biomedicale des Armees, Bretigny-sur-Orge, France

**Keywords:** Pancytopenia, Gene expression, miRNA, Hematological acute radiation syndrome (HARS)

## Abstract

Based on gene expression changes measured in the peripheral blood within the first 2 days after irradiation, we predicted a pancytopenia in a baboon model. Eighteen baboons were irradiated with 2.5 or 5 Gy. According to changes in blood cell counts, the surviving baboons (*n* = 17) exhibited a hematological acute radiation syndrome (HARS) either with or without a pancytopenia. We used a two stage study design where stage I was a whole genome screen (microarrays) for mRNA combined with a qRT-PCR platform for simultaneous detection of 667 miRNAs using a part of the samples. Candidate mRNAs and miRNAs differentially upregulated or downregulated (>2-fold, *p* < 0.05) during the first 2 days after irradiation were chosen for validation in stage II using the remaining samples and using throughout more sensitive qRT-PCR. We detected about twice as many upregulated (mean 2128) than downregulated genes (mean 789) in baboons developing an HARS either with or without a pancytopenia. From 51 candidate mRNAs altogether, 11 mRNAs were validated using qRT-PCR. These mRNAs showed only significant differences between HARS groups and H0, but not between HARS groups with and without pancytopenia. Six miRNA species (e.g., miR-574-3p, *p* = 0.009, ROC = 0.94) revealed significant gene expression differences between HARS groups with and without pancytopenia and are known to sensitize irradiated cells. Hence, in particular, the newly identified miRNA species for prediction of pancytopenia will support the medical management decision making.

**Electronic supplementary material:**

The online version of this article (doi:10.1007/s00277-017-2952-7) contains supplementary material, which is available to authorized users.

## Introduction

In a large-scale radiological emergency, early detection of exposed individuals would be required in order to evaluate the extent of radiation injuries and, when needed, decide in favor of a hospitalization and assign an appropriate treatment [1–3]. In particular, after high-dose exposure (≥2 Gy single whole body dose), severe acute health effects (acute radiation syndrome, ARS) will occur and early diagnosis within 1–3 days after exposure is pivotal to hospitalize exposed individuals in specialized clinics and to start the appropriate treatment as soon as possible.

In approaches like MEdical TREatment ProtocOLs (METREPOL), early detected clinical signs and symptoms are used for prediction of the late occurring hematologic ARS (HARS, [4]). METREPOL categorizes HARS into four severity degrees (H1–4) based on blood cell count (BCC) changes in the weeks that follow the exposure: no HARS (H0), low (H1), medium (H2), severe (H3), and fatal (H4) HARS. With the decrease in neutrophils and platelets in the peripheral blood, the hematological syndrome of the ARS is characterized mainly by immune suppression and hemorrhage over time. We hypothesized that the depletion of BCC would be preceded by changes in gene expression causally or timely related to their later decline and, therefore, could serve as an early indication of late occurring HARS severity score.

In previous studies, we successfully identified certain messenger RNAs (mRNAs) and microRNAs (miRNAs) predicting the late occurring HARS severity [5, 6]. However, when using METREPOL, we often experienced difficulties in the categorization, since, e.g., neutrophil counts during follow-up could reflect an H2 while platelets appeared more representative of an H1 degree HARS. However, medical management decisions for H1 (e.g., no hospitalization required) differ considerably from H2 (hospitalization and active supportive care required). As a result, we ultimately merged categories and came up with, e.g., H1–2 or H2–3 degree HARS, which adds additional categories to the four HARS severity categories according to METREPOL. Communications with clinicians confirmed the view that the prediction of patients developing a clinical relevant HARS degree either without or with a pancytopenia would be the most relevant categories regarding medical management decision making. Hence, we simplified the current study and searched for gene expression changes, namely mRNA and miRNAs, within the first 2 days after exposure in order to predict a clinical relevant HARS associated with or without a pancytopenia.

In collaboration with the French Army Biomedical Research Institute, we assessed blood samples obtained from irradiated baboons taken before (day 0) and 1 and 2 days after partial/total body exposure. BCC were measured in these baboons during the entire follow-up period in order to detect a clinical significant HARS associated either with or without pancytopenia. Pancytopenia was defined as a reduced number of neutrophils <500/μl over ≥10 days combined with platelets ≤10,000/μl measured at least once during the follow-up and a reduced number of red blood cells corresponding to an Hb ≤ 8 g/dl measured at least once during the follow-up [7, 8]. On the blood samples taken before and 1–2 days after exposure, we performed a whole genome screening and identified protein-coding mRNA genes associated with late occurring clinical relevant HARS with and without pancytopenia. These mRNAs were then validated using qRT-PCR. We also screened for 667 miRNAs using a qRT-PCR platform. The selected candidate miRNAs were also validated on the remaining samples in stage II using the same qRT-PCR platform but restricting the analysis on the candidate miRNAs from stage I.

## Materials and methods

### Animals

Eighteen baboons were bred by the Centre National de la Recherche Scientifique (Rousset sur Arc, France) for the purpose of biomedical research. In the nonhuman primate facility of the French Army Biomedical Research Institute, the baboons were placed in individual cages at 21 °C, with a relative humidity of 55% and a 12-/12-h light-dark schedule. The animals received fresh fruit and solid food twice a day and had access to water ad libitum. The male baboons had an average age of 8.1 years (±3.3 years) and weighed 23.7 (±5.2 kg). The experiment was approved by the French Army Animal Ethics Committee (no. 2010/12.0). All baboons were treated in compliance with the European legislation related to animal care and protection in order to minimize pain and damage. The total number of baboons evaluated in this study decreased to 17, for reasons described below.

### Irradiation

The animals were anesthetized with a combination of tiletamine and zolazepam (6 mg kg^−1^ intramuscularly, Zoletil 100; Virbac, Carros, France) before irradiation. Then, the baboons were placed in restraint chairs, sitting orthogonally, front to a horizontal and homogeneous field of gamma rays delivered by a ^60^Co source (IRDI 4000; Alsthom, Levallois, France) to perform either total body irradiation (TBI) or partial body irradiation (PBI). In order to attain different patterns of PBI, a 20-cm thick lead screen was used to shield different parts of the body as detailed in Table 1. Two baboons were exposed to 5 Gy TBI and two others to 2.5 Gy TBI. Eight different exposure patterns were simulated and two baboons were exposed per pattern which summed up to 16 baboons receiving PBI (for details, see ref. [9]; Table 1) corresponding to an equivalent TBI dose of 2.5 or 5 Gy. Two dose rates were used (8 cGy/min for 5 Gy TBI and 5 Gy 50% PBI and 32 cGy/min for all other situations) because the Cobalt 60 source was changed during this study. Moreover, to achieve the same homogeneous radiation field whatever the dose rate, all baboons were irradiated at the same distance from the source. Consequently, radiation exposures lasted between 8 and 62 min. The midline tissue (right anterior iliac crest) dose in air was measured with an ionization chamber. Delivered doses were controlled by alumina powder thermoluminescent dosimeters placed on different cutaneous areas (thorax, thoracic and lumbar vertebrae, head, tibia, femur, femoral head; for details, see ref. [9]).

**Table 1:** Exposure pattern and corresponding effect

	Exposure (Co-60)				Screening (stage I) mRNA and miRNA			Validation (stage II, all available samples)					
								mRNA			miRNA		
ID no.	Radiation dose (Gy, free in air)	PBI/TBI	Days after exposure	HARS degree	H0	HARS with pancytopenia	HARS without pancytopenia	H0	HARS with pancytopenia	HARS without pancytopenia	H0	HARS with pancytopenia	HARS without pancytopenia
1	5 PBI 50%	Left-hemibody exposed	0	0	0 day			0 day					
			1	2						1 day			1 day
			2	2						2 days			2 days
2	5 PBI 50%	Left-hemibody exposed	0	0	0 day			0 day			0 day		
			1	2						1 day			1 day
			2	2						2 days			2 days
3	15 PBI 30%	Head + arms exposed	0	0	0 day			0 day			0 day		
			1	1–2						1 day			1 day
			2	1–2						2 days			2 days
4	15 PBI 30%	Head + arms exposed	0	0	0 day			0 day			0 day		
			1	1–2						1 day			1 day
			2	1–2									2 days
5	6.25 PBI 80%	2 legs shielded	0	0				0 day			0 day		
			1	2–3						1 day			1 day
			2	2–3									2 days
6	6.25 PBI 80%	2 legs shielded	0	0				0 day			0 day		
			1	2			1 day						1 day
			2	2			2 days						2 days
7	10 PBI 50%	Left-hemibody exposed	0	0				0 day			0 day		
			1	1–2						1 day			1 day
			2	1–2						2 days			2 days
8	10 PBI 50%	Left-hemibody exposed	0	0				0 day			0 day		
			1	1–2			1 day						1 day
			2	1–2			2 days						2 days
9	5.55 PBI 90%	1 leg shielded	0	0				0 day			0 day		
			1	3									1 day
			2	3									2 days
10	5 TBI	TBI	0	0	0 day			0 day			0 day		
			1	2			1 day						1 day
			2	2			2 days						2 days
11	7.5/2.5 TBI	TBI	0	0				0 day			0 day		
			1	3			1 day						1 day
			2	3			2 days			2 days			2 days
12	5.55 PBI 90%	1 leg shielded	0	0				0 day			0 day		
			1	2			1 day			1 day			1 day
			2	2			2 days			2 days			2 days
13	6.25 PBI 80%	2 legs shielded	0	0				0 day			0 day		
			1	2		1 day						1 day	
			2	2		2 days						2 days	
14	6.25 PBI 80%	Head neck shielded	0	0				0 day			0 day		
			1	2		1 day			1 day			1 day	
			2	2		2 days			2 days			2 days	
15	2.5 TBI	TBI	0	0				0 day			0 day		
			1	2–3		1 day			1 day			1 day	
			2	2–3		2 days						2 days	
16	2.5 TBI	TBI	0	0				0 day			0 day		
			1	2–3		1 day						1 day	
			2	2–3		2 days						2 days	
17	5 TBI	TBI	0	0				0 day			0 day		
			1	2		1 day			1 day			1 day	
			2	2		2 days			2 days			2 days	
					0 day #5	1 day #5; 2 days #5	1 day #5; 2 days #5	0 day #17	1 day #3; 2 days #2	1 day #7; 2 days #6	0 day #16	1 day #5; 2 days #5	1 day #12; 2 days #12

The left part of the table summarizes radiation exposure scenarios and resulting hematologic acute radiation syndrome (HARS) severities and a time scale with days after irradiation. Partial body irradiation (PBI) and total body irradiation (TBI) were performed, and details on PBI are summarized below the subtitle. The TBI 7.5/2.5 Gy represents a sequential protocol of irradiation using 2.5 Gy TBI at first followed by an additional 5 Gy exposure with hemibody shielding. Exposure between the two fractions was stopped for 5 min. The right part of the table shows blood samples used for screening at stage I followed by the blood samples used for a validation of mRNA and miRNA species (using all available blood samples including those remaining from stage I). The total number of blood samples used for stage I and stage II are summed up on the bottom of each column

### Blood collection, determination of HARS severity scores, and pancytopenia

Using changes in BCC observed days to weeks after irradiation, the severity scores (0, unexposed, 1–4, low-severe degree) of the HARS could be determined following METREPOL [4]. Often, changes in lymphocyte counts or platelets over time indicated HARS degrees differing from each other so that intermediates between, e.g., HARS 2 and 3 had to be defined. Pancytopenia was identified based on a reduced number of neutrophils (<500/μl over >10 days), platelets (<10,000/μl), and red blood cells (Hb < 8 g/dl) [7, 8]. Reduced numbers of platelets and red blood cells had to be measured at least once during the follow-up. The HARS score as well as pancytopenia was based on changes in differential blood counts taken at up to 22 time points over the course of 7–203 days after exposure. Of note, whole blood samples for gene expression measurements were taken only before irradiation (0 h) and at 1 and 2 days after irradiation in order to predict late occurring HARS using radiation induced changes in gene expression preceding the development of HARS.

### RNA extraction and quality control

Whole blood samples (2.5 ml) were processed following the PAXgene Blood RNA system (BD Diagnostics, PreAnalytiX GmbH, Hombrechtikon, Switzerland). In brief, blood was drawn into a PAXgene Blood RNA tube at the French Army Biomedical Research Institute. The tube was gently inverted (10 times) and stored at room temperature overnight then at −20°. After all samples were collected, the PaxGene tubes were sent to Germany for further processing. After thawing, washing, and centrifugation, cells in the supernatant were lysed (proteinase K) followed by addition of lysis/binding solution taken from the mirVana Kit (Life Technologies, Darmstadt, Germany). With the mirVana kit, total RNA, including small RNA species, was isolated by combining a phenol-chloroform RNA precipitation with further processing using a silica membrane. After several washing procedures, DNA residuals became digested on the membrane (RNAse free DNAse Set, Qiagen, Hilden, Germany). RNA was eluted in a collection tube and frozen at −20 °C. Quality and quantity of isolated total RNA were measured spectrophotometrically (NanoDrop, PeqLab Biotechnology, Erlangen, Germany). RNA integrity was assessed by the 2100 Agilent Bioanalyzer (Life Science Group, Penzberg, Germany), and DNA contamination was controlled by conventional PCR using an actin primer. We used only RNA specimens with a ratio of A_260_/A_280_ ≥ 2.0 (Nanodrop) and RNA integrity number (RIN) ≥ 7.5 for whole genome microarray (IMGM Laboratories, Martinsried, Germany) or RIN ≥ 7.3 for qRT-PCR analyses.

### Stage I screening: whole genome microarray

The whole genome screening for differentially expressed genes (DEG) (protein-coding mRNAs) was performed on 25 RNA samples with a subsequent range of HARS scores (H0 *n* = 5; HARS without pancytopenia *n* = 2 × 5, on days 1 and 2 after exposure; HARS with pancytopenia *n* = 2 × 5, on days 1 and 2 after exposure; online resource 1). We used the Agilent oligo microarray GE 8x60K (Agilent Technologies, Waldbronn, Germany) combined with a one-color-based hybridization protocol of GeneSpring GX12 software for data analysis as described in detail elsewhere [10]. We analyzed gene expression by quantile normalized log_2_-transformed probe signals as an outcome. We used the nonparametric Mann-Whitney (MW) test to compare gene expression across HARS with and without pancytopenia groups using the unexposed group (H0) as the reference (control). Only those gene transcripts that had a call “present” in at least 60% of RNA specimens were included in the analysis of gene expression, and only genes with MW *p* values ≤0.05 and a >2-fold gene expression difference among compared groups were considered to represent a candidate gene for validation in stage II. Due to the explorative nature of this study, the low sample size and the nonparametric statistics employed, we did not correct for multiple comparisons on the screening stage I of the study but considered this within our bioinformatic approach as well as the validation stage II of our study where the numbers of hypotheses tested in parallel becomes reduced from about 20,000 (stage I) to 51 mRNAs and 23 miRNAs in stage II (see below). Gene expression data presented in this publication have been deposited at the NCBI’s Gene Expression Omnibus (GEO accession number GSE77254).

### Bioinformatics

All genes associated with *p* values ≤0.05 and a >2-fold gene expression difference (up or down) relative to the reference underwent gene set enrichment analyses using PANTHER pathway software (http://www.pantherdb.org/, version 10.0). PANTHER groups genes with similar biological function based on their annotation (reference list was the current *Homo sapiens* GO database). For these *p* values, we corrected for multiple testing by employing the Bonferroni algorithm.

### Stage II: validation of stage I candidate genes via qRT-PCR

For validating the mRNA candidate genes from stage I (screening) using remaining RNA samples (online resource 1), we used a custom low density array (LDA; high-throughput qRT-PCR platform) and TaqMan chemistry. A 1-μg RNA aliquot of each RNA sample was reverse transcribed using a two-step PCR protocol (High Capacity Kit). Four hundred microliters of cDNA (1 μg RNA equivalent) was mixed with 400 μl 2× RT-PCR master mix and pipetted into the eight fill ports of the LDA. Cards were centrifuged twice (1200 rpm, 1 min, Multifuge 3S-R, Heraeus, Germany), sealed, and transferred into the 7900 qRT-PCR instrument. The qRT-PCR was run for 2 h following the qRT-PCR protocol for 384-well LDA format. All measurements were run in duplicate.

A commercially available 384-well LDA was used that provided the simultaneous detection of 380 different miRNAs. Two different LDAs (type A and B) were combined so that the detection of 667 miRNA species (partly spotted in duplicate to completely fill the LDA) was possible. Aliquots from each RNA sample (in general 2 μg total RNA/LDA type A/B) were reversely transcribed without preamplification over 3 h using “Megaplex pools without preamplification l for microRNA expression analysis protocol.” Using different sets of primers, two kinds of cDNAs suitable for each of both LDAs were created. In a second step, the whole template cDNA and 450 μl 2× RT-PCR master mix were adjusted to a total volume of 900 μl by adding nuclease free water, and aliquots of 100 μl were pipetted into each fill port of a 384-well human LDA. Cards were centrifuged twice (see above), sealed, and transferred into the 7900 RTQ-PCR instrument and again, the 384-well LDA RTQ-PCR protocol was run over 2 h.

All technical procedures for qRT-PCR were performed in accordance with standard operating procedures implemented in our laboratory in 2008 when the Bundeswehr Institute of Radiobiology became certified according to DIN EN ISO 9001/2008. All chemicals for qRT-PCR using TaqMan chemistry were provided by Life Technologies, Darmstadt, Germany.

For the custom LDA, CT values were normalized relative to the 18S ribosomal RNA (rRNA) measured in an aliquot of the RNA samples using a 96-well format TaqMan qRT-PCR platform. We have found that this approach to normalization was more robust compared to the use of the internal control (GAPDH and 18S rRNA) spotted on the LDA. For the commercial LDA, we used the median miRNA expression on each LDA for normalization purposes, because this proved to be the more robust and slightly more precise method compared to a normalization approach using a housekeeping miRNA species provided on the LDA (data not shown). The CT values of the housekeeping gene was subtracted from the CT value of each of the spotted genes, following the ∆CT—quantitative approach for normalization purposes.

### Statistical analysis

Using the quantitative gene expression results from stage II, we examined none (H0) vs HARS groups with and without pancytopenia and we compared HARS groups with each other. Descriptive statistics (*n*, mean, standard deviation, min, max) and *p* values (*t* test and the nonparametric Kruskal-Wallis test (KW), where applicable) were calculated for each of the variables (candidate mRNAs and miRNAs) and per time point. Logistic regression analysis was performed on binary outcome variable for each of the variables (genes) of interest separately (univariate). Binary outcome variables comprised comparisons of either HARS groups relative to the unexposed H0 group or between HARS groups with and without pancytopenia. Odds ratios (OR), 95% confidence intervals (95% CI), and corresponding *p* values (Wald chi-square) were calculated. We also determined the area under a receiver-operator characteristic (ROC) curve providing a reasonable indication of overall diagnostic accuracy. ROC areas of 1.0 indicate complete agreement between the predictive model and the known HARS group and thus a clear distinction between healthy (H0) animals and baboons’ subsequently showing clinically relevant HARS with or without pancytopenia. All calculations were performed using SAS (release 9.2, Cary, NC, USA).

## Results

### Material available for the two-stage study design

Due to unusual blood cell counts before irradiation and a sudden death after irradiation, one out of the 18 baboons had to be excluded, leaving 17 baboons eligible for analysis.

During the *screening approach at stage I*, we assessed 25 whole genome microarrays for 25 blood samples (Table 1, online resource 1). Blood samples collected before irradiation from five baboons were selected randomly and represented H0 degree HARS (*n* = 5). Five baboons developed an HARS with pancytopenia and five blood samples were selected on day 1 and day 2 after irradiation for screening purposes (*n* = 10). Ten blood samples from another five baboons developing a clinically relevant HARS without pancytopenia were chosen randomly on the first 2 days after irradiation (*n* = 10). The same blood samples were used for screening of 667 miRNAs employing a commercially available LDA.

For the *validation of mRNA and miRNAs at stage II*, we used all available blood samples irrespective of whether they were already used for screening purposes. For examinations of mRNAs, the sample numbers were 17, 5, and 13 for H0, HARS with pancytopenia (3 samples on day 1 and 2 samples on day 2 after irradiation), and clinically relevant HARS without pancytopenia (7 samples on day 1 and 6 samples on day 2 after irradiation), respectively (Online resource 1; Table 1). For examinations of miRNAs altogether, 50 samples were utilized comprising H0 (*n* = 16), HARS with pancytopenia (5 samples on days 1 and 2 after irradiation, total *n* = 10), and clinically relevant HARS without pancytopenia (12 samples on days 1 and 2 after irradiation, *n* = 24).

### Identification of HARS with and without pancytopenia

**Fig. 1:**
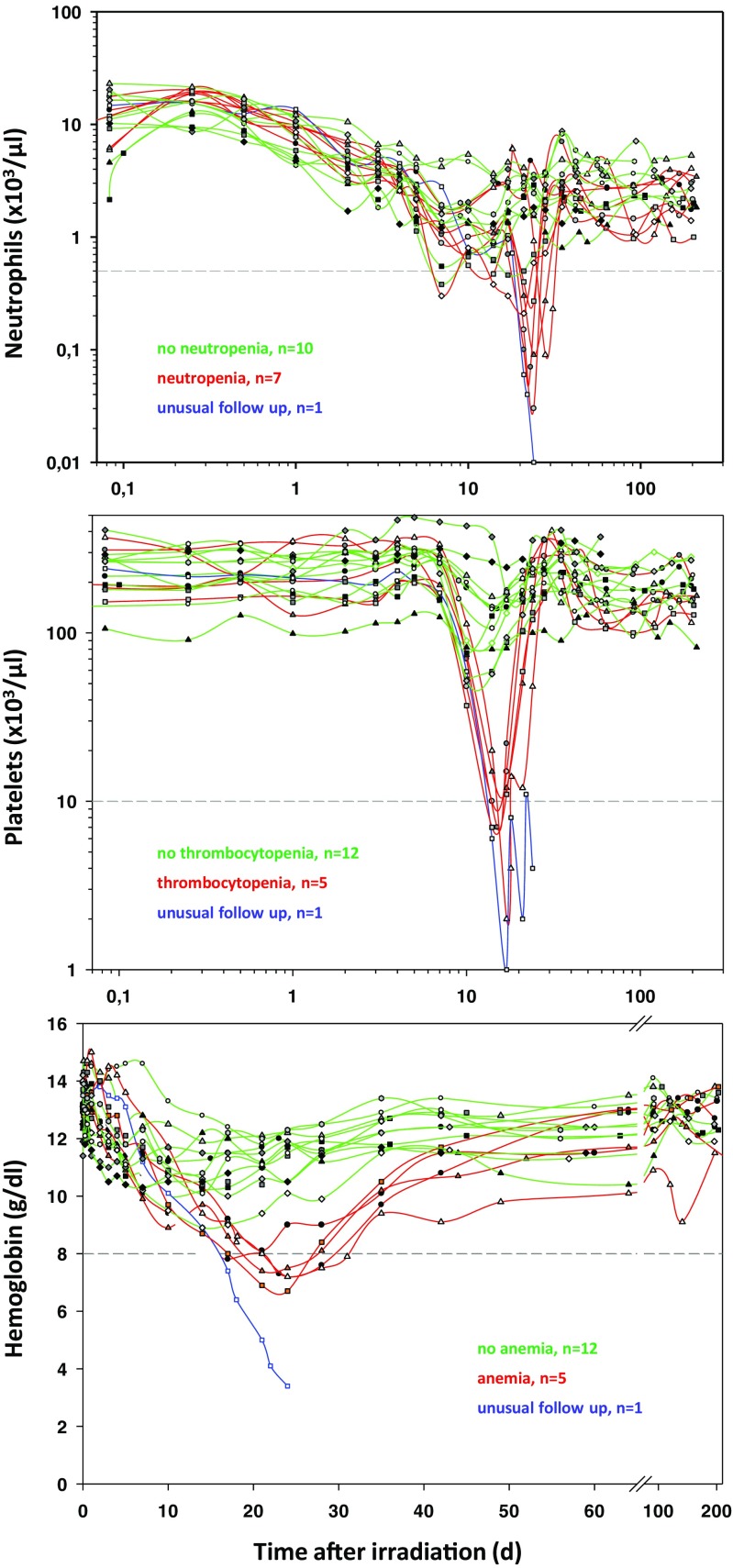
Changes in blood cell counts of neutrophils (*upper graph*), platelets (*middle graph*), and red blood cells (hemoglobin, *lower graph*) are shown for all 18 baboons up to 203 days after exposure. HARS severity was determined separately for count changes in neutrophils, lymphocytes, and platelets during the whole follow-up starting at day 7. *Gray dashed lines* indicate limits (neutrophils: 0.5 × 1000/μl; platelets, 10 × 1000/μl; red blood cells/hemoglobin, 8 g/dl) for the definition of a pancytopenia

Changes in blood cell counts were observed over up to 202 days after irradiation. A decline in neutrophils, platelets and red blood cells was observed (Fig. 1). Based on the METREPOL definition for HARS and our criteria for pancytopenia (see above) we identified HARS with pancytopenia (red lines) and clinically relevant HARS without pancytopenia (green lines).

### Stage I: RNA isolation and whole genome microarray results

From 2.5 ml whole blood, we isolated 10, 8.5, and 6.3 μg total RNA on average before irradiation and 1 and 2 days after irradiation, respectively. RNA integrity (RIN) with a mean value of 8.6 (stdev ±0.6, min 7.3, max 9.5) suggested high-quality RNA sufficient for running both methods.

**Fig. 2:**
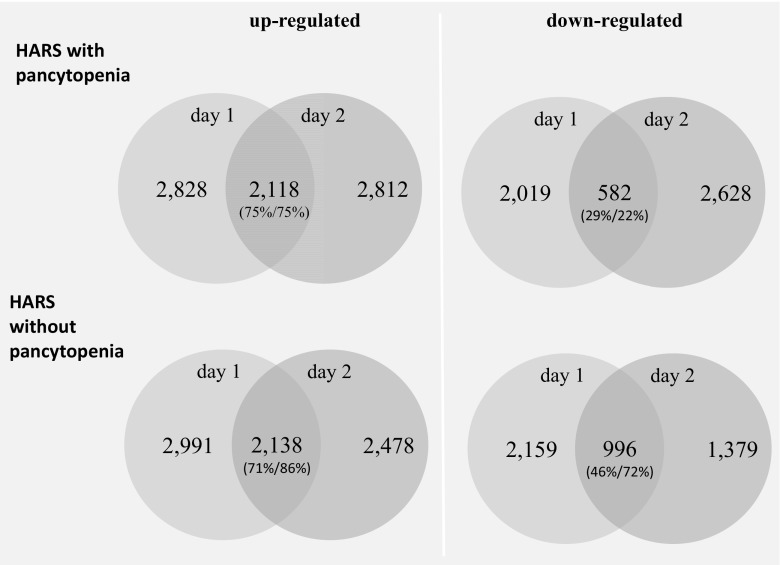
Venn diagrams showing the number of upregulated (*left side*) and downregulated (*right side*) protein coding genes (mRNA transcripts) observed for HARS with pancytopenia and HARS without pancytopenia. Differentially expressed genes (DEG) observed on both days after exposure are shown in the *overlapping circle*. *Numbers* outside the overlapping region represent the total number of differentially expressed genes that were not in common over day 1 to day 2. *Percentages in parenthesis* refer to the number of overlapping genes relative to the DEG of day 1 (first entry in *parenthesis*) and day 2 (second entry in *parenthesis*)

From about 20,000 protein-coding mRNAs, 46% on average (range: 34–54%) appeared expressed. An about equal number of 2000–2800 upregulated and downregulated DEG was observed on both days after irradiation in HARS either with or without pancytopenia (Fig. 2). As an exception, only 1379 DEG were observed at day 2 for HARS without pancytopenia. The overlapping number of DEG over both days was in the range of 71–86% for the upregulated genes and lower (22–29% for HARS with pancytopenia and 46–72% for HARS without pancytopenia) for the downregulated genes.

For the bioinformatic approach using PANTHER, at least 100 protein-coding genes (mRNAs) as input data are required. Therefore, PANTHER could be performed for the overlapping number of upregulated/downregulated mRNAs over both days and separately for HARS groups with and without pancytopenia (Table 2). Messenger RNAs coding for many biological processes (e.g., immune response or cell communication), protein classes (e.g., cytokine receptors), molecular functions (e.g., protein, RNA, or nucleic acid binding), and pathways (e.g., inflammation mediated by chemokine/cytokines or Toll receptor signaling) appeared overrepresented in HARS irrespective of whether a pancytopenia was developed or not. However, additional overrepresented numbers of mRNAs were observed for HARS with pancytopenia regarding biological processes (e.g., macrophage activation or protein phosphorylation), molecular functions (ion channel activity), and pathways namely the integrin and the apoptosis signaling pathways (bold entries, Table 2). For HARS without pancytopenia, additional overrepresented numbers of mRNAs were coding for biological processes or protein classes and were involved in mRNA processing or ribosomal proteins.

**Table 2:** PANTHER classification for the HARS groups with and without pancytopenia

PANTHER classification	HARS without pancytopenia			HARS with pancytopenia		
	Upregulated/downregulated	Over/under repres.	*p* values	Upregulated/downregulated	Over/under repres.	*p* values
Biological process						
B cell-mediated immunity	Down/up	++	1.9E−06/1.3E−07	Down/up	++	5.2E−05/3.5E−07
Natural killer cell activation	Up	+	4.3E−08	Down/up	++	2.6E−04/3.1E−07
Immune response	Down/up	++	3.4E−04/4.6E−11	Up	+	3.3E−09
Immune system process	Up	+	1.9E−07	Up	+	5.0E−08
Cell communication	Up	+	9.4E−06	Up	+	8.0E−08
Cellular process	Up	+	5.7E−07	Up	+	4.0E−07
Response to stimulus	Up	+	7.6E−06	Up	+	5.9E−06
Translation	Down/up	+−	5.5E−04/5.1E−06	Up	−	1.0E−05
Cell death	Up	+	5.7E−05	Up	+	3.4E−05
Apoptotic process	Up	+	5.7E−05	Up	+	3.4E−05
Death	Up	+	6.3E−05	Up	+	3.7E−05
mRNA processing	*Up*	*−*	*3.8E−05*			
RNA metabolic process	*Up*	*−*	*8.5E−05*			
Organelle organization				*Down*	*+*	*3.7E−04*
Carbohydrate metabolic process				*Down*	*-*	*7.1E−04*
Macrophage activation				*Up*	*+*	*2.5E−05*
Protein phosphorylation				*Up*	*+*	*6.2E−05*
Regulation of catalytic activity				*Up*	*+*	*9.7E−05*
Protein class						
Cytokine receptor	Up	+	8.6E−08	Down/up	++	3.6E−04/4.7E−07
Enzyme modulator	Up	+	4.9E−05		+	3.0E−06
RNA binding protein	Up	−	2.9E−08		−	8.9E−06
G-protein modulator	Up	+	1.8E−05		+	8.0E−05
Ribosomal protein	*Down*	*+*	*9.0E−09*			
Nucleic acid binding	*Up*	*−*	*4.9E−05*			
Defense/immunity protein	*Up*	*+*	*4.6E−06*			
Immunoglobulin receptor superfamily	*Up*	*+*	*7.3E−05*			
mRNA processing factor	*Up*	*−*	*9.6E−05*			
Molecular function						
Protein binding	Up	+	8.4E−09	Up	+	2.3E−11
RNA binding	Up	−	8.5E−08	Up	−	1.7E−06
Nucleic acid binding	Up	−	2.1E−05	Up	−	2.8E−05
Small GTPase regulator activity	Up	+	4.1E−06	Up	+	4.9E−05
Structural constituent of ribosome	*Down*	*+*	*3.2E−06*			
mRNA binding	*Up*	*−*	*3.5E−05*			
Ion channel activity				*Down*	*−*	*4.2E−04*
Pathway						
Inflammation mediated by chemokine and cytokine signaling pathway	Up	+	4.5E−07	Up	+	1.5E−09
Toll receptor signaling pathway	Up	+	4.2E−05	Up	+	1.1E−07
Pentose phosphate pathway	*Up*	*+*	*6.6E−05*			
Integrin signaling pathway				*Up*	*+*	*5.3E−05*
Apoptosis signaling pathway				*Up*	*+*	*4.6E−04*

Using the overlapping number of DEG from day 1 and day 2 after exposure for HARS groups with and without pancytopenia, a classification of overrepresented and underrepresented genes coding, e.g., biological processes or protein classes, was conducted using the bioinformatic tool PANTHER (http://www.pantherdb.org; version 10.0) which comprises Gene Ontology (GO) annotations directly imported from the GO database. Based on the comparison of observed vs expected numbers of upregulated or downregulated genes (reference database was *Homo sapiens*) for biological processes, e.g., “immune system process,” an overrepresentation (+) or underrepresentation (−) in the number of genes annotated to this process and a corresponding *p* value (Bonferroni corrected) was calculated. Numbers in italics refer to processes which differ among both HARS groups

Based on the fold difference, the *p* value, and a preferable sustained changed mRNA expression over the 2 days after irradiation, we aimed to select candidate mRNAs for validation at stage II. Despite the high overlap in DEG over time (Fig. 2), we found no satisfying DEG being similarly expressed at both days. Also, all DEG of interest were differentially expressed in HARS with or without pancytopenia relative to H0. However, we experienced up to 6-fold differences in DEG in blood samples from baboons suffering from HARS with pancytopenia relative to HARS without pancytopenia. Using these prerequisites, we selected 51 candidate mRNAs (36 mRNAs for day 1 and 15 mRNAs for day 2) and forwarded them for validation in stage II using qRT-PCR.

During the screening of 667 miRNAs, we identified 23 miRNAs showing significant DEG of HARS with pancytopenia vs clinically relevant HARS without pancytopenia on days 1 (*n* = 17) and day 2 (*n* = 6) with two miRNAs (miR-584, miR-1290) overlapping on both days.

### Stage II: validation using qRT-PCR measurements

During stage II validation of the 51 candidate mRNAs from stage I, 28 mRNAs showed either no amplification plot or amplification plots in a minority of all samples (≤3). Those were excluded from further analysis. Twelve genes revealed no significant changes in gene expression in baboons developing an HARS relative to H0 using qRT-PCR. There remained nine genes for identification of HARS with or without pancytopenia (Table 3) relative to H0 for the first day after exposure and two genes for the second day after exposure. Most of the genes from day 1 appeared 2-fold downregulated (e.g., *CDCA7L* or *GBP2*), but three were 3–5-fold upregulated (*C11orf96*, *GLUL*, *TM4SF19*) relative to H0 (Table 3) when developing a HARS without pancytopenia. For the second day, two genes appeared 2–3-fold downregulated when developing a HARS without pancytopenia. These fold differences increased up to 2-fold when developing an HARS with pancytopenia but did not become statistically significant (Table 3).

**Table 3:** Summary of validated qRT-PCR results

	H0 (unexposed control)					HARS without pancytopenia							HARS with pancytopenia							HARS with vs without pancytopenia	
Gene ID	*n* (17/16)	Mean	Stdev	Min	Max	*n* (7/12)	Mean	Stdev	Min	Max	FC	ttest/KW	*n* 3/5)n	Mean	Stdev	Min	Max	FC	ttest/KW	FC	ttest/KW
*First day after exposure*																					
C11orf96	14	18.7	1.0	17.0	20.2	5	17.0	1.1	15.9	18.4	3.3	0.005*	3	16.4	1.6	14.7	17.9	4.9	0.0056*	1.5	0.6
CDCA7L	17	16.2	0.8	14.5	17.5	7	17.1	1.3	15.5	19.4	0.5	0.04	3	18.4	1.9	17.1	20.6	0.2	0.2	0.4	0.2
GBP2	15	16.8	1.3	14.7	19.0	5	17.7	1.4	15.7	19.6	0.5	0.2	3	18.4	1.0	17.4	19.2	0.3	0.05	0.6	0.5
GLUL	15	9.9	0.7	8.7	11.1	5	8.5	0.5	7.6	9.0	2.6	0.001*	3	8.2	0.5	7.8	8.8	3.2	0.002*	1.2	0.5
HERC5	17	16.7	1.1	15.1	18.5	7	17.9	1.8	16.0	21.2	0.5	0.2	3	18.5	0.5	18.0	18.9	0.3	0.02	0.6	0.6
HMHA1	15	14.6	0.5	13.8	15.5	5	14.3	0.1	14.2	14.5	1.3	0.02	3	14.3	0.3	14.1	14.7	1.3	0.3	1.0	0.9
PPP3CC	17	14.5	0.5	13.2	15.2	7	15.2	0.6	14.4	16.1	0.6	0.008	3	15.4	0.4	15.0	15.8	0.5	0.01	0.9	0.7
TIMM10B	16	16.2	0.5	15.2	17.2	6	16.9	0.7	16.0	18.2	0.6	0.02	3	17.1	0.5	16.5	17.6	0.5	0.01	0.9	0.7
TM4SF19	5	19.7	0.7	18.6	20.4	6	17.3	1.1	15.5	18.3	5.3	0.002*	2	16.5	1.1	15.7	17.3	9.4	0.005*	1.8	0.4
miR-124	9	18.9	0.8	17.1	19.8	9	19.4	0.3	18.9	19.8	0.7	0.09	3	18.6	0.2	18.3	18.8	1.2	0.6	1.7	0.002*
miR-146a	16	13.1	0.6	12.2	14.2	11	14.5	0.5	13.7	15.7	0.4	<0.0001*	4	14.0	0.3	13.7	14.4	0.5	0.0076*	1.4	0.1
miR-29c	15	18.1	0.9	16.2	19.2	10	18.8	0.9	17.7	19.8	0.6	0.07	5	17.6	1.0	16.0	18.5	1.4	0.3	2.3	0.03
miR-378	16	10.9	0.8	9.7	12.8	10	11.3	1.3	9.1	13.8	0.7	0.3	5	10.4	0.3	9.8	10.7	1.4	0.2	1.9	0.05
miR-574-3p	16	11.6	0.6	10.6	12.5	11	11.4	0.3	10.9	11.7	1.1	0.9	4	10.5	0.6	9.8	11.2	2.1	0.003*	2.0	0.009
rno-miR-7#	16	14.0	0.9	12.6	15.3	10	14.6	0.6	13.5	15.6	0.7	0.1	5	13.2	0.8	12.1	14.0	1.7	0.1	2.6	0.003*
*Second day after exposure*																					
CDCA7L	17	16.2	0.8	14.5	17.5	6	17.7	1.3	15.6	19.1	0.3	0.002*	2	18.9	0.4	18.6	19.1	0.2	0.0002*	0.5	0.3
PPP3CC	17	14.5	0.5	13.2	15.2	6	15.6	0.6	14.7	16.2	0.5	0.0004*	2	15.6	0.4	15.4	15.9	0.5	0.01*	1.0	1.0
miR-584	16	16.1	0.7	14.8	17.2	8	17.3	0.7	16.2	18.4	0.4	0.0002*	4	16.8	0.4	16.4	17.2	0.6	0.06	1.5	0.2
miR-720	16	6.5	0.6	5.4	8.0	11	7.4	0.9	5.7	8.7	0.6	0.01*	4	6.7	0.4	6.2	7.0	0.9	0.6	1.5	0.2
miR-133a	16	16.7	1.3	14.6	19.3	10	16.3	1.8	13.1	18.5	1.3	0.6	5	14.3	1.0	12.6	15.2	5.1	0.002*	4.1	0.04

The table summarizes validated qRT-PCR results (normalized threshold cycle [CT] values) of candidate genes from stage I examined in H0 (day 0 after exposure) and in HARS without and with pancytopenia on days 1 and 2 after radiation exposure. Descriptive statistics reflect the distribution and the fold change (FC) difference among genes differentially expressed relative to H0 as the reference and between both HARS groups in order to compare discrimination between HARS without and with pancytopenia. Numbers in parenthesis for columns entitled *n* reflect the number of sample where gene expression measurements could be successfully performed. The first and the second number in parenthesis provide the total sample size used for mRNA and miRNA measurements, respectively. The numbers shown per gene can be smaller compared to the total number of samples examined per group (see Table 1). Where applicable, either the *t* test (ttest) or the Kruskal-Wallis (KW) test was performed for group comparisons. The *p* values corrected for multiple comparisons (Bonferroni) are marked with an asterisk when they reach values such as *p* < 0.006 (*p* = 0.05/9 genes/hypothesis) and *p* < 0.008 (*p* = 0.05/6 genes/hypothesis) for mRNA and miRNAs after first day and *p* < 0.025 (*p* = 0.05/2 genes/hypothesis) and *p* < 0.017 (*p* = 0.05/3 genes/hypothesis) for mRNA and miRNAs after the second day of exposure

**Fig. 3:**
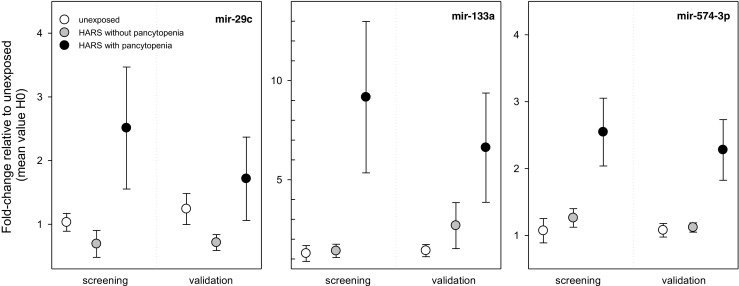
Gene expression changes in the peripheral blood were examined in baboon groups being either unexposed (H0) or belonging to HARS groups with and without pancytopenia. We plotted the fold change (FC) of candidate genes from the screening stage relative to the mean H0 values on day 1 (miR-29c and miR-574-3p) and day 2 (miR-133a) after exposure. FCs of the genes from the screening stage are shown at the *left side*, and corresponding data from the validation stage are shown on the *right side* of the graphs. The validation stage includes additional samples not used during the screening stage I (see also Table 1). *Symbols* represent mean values and *error bars* reflect the SEM. The number of measurements per group is shown in Table 3

Examinations on miRNAs on day 1 after exposure showed five miRNA species (miR-124, miR-29c, miR-378, miR-574-3p, and rno-miR-7#) with significantly 1.7–2.6-fold higher mean DEG in baboons developing a HARS with pancytopenia vs those developing a clinically relevant HARS without pancytopenia (Table 3). For the second day, in particular, miR-133a appeared promising due to a 4.1-fold increased mean DEG for HARS with pancytopenia in comparison to HARS without pancytopenia (Table 3). The discrimination of HARS groups during screening using, e.g., miR-29c or miR-133 became reduced during the validation step (Fig. 3): Either the gene expression values of the unexposed group (miR-29C) or the HARS group without pancytopenia (miR-133) reached gene expression values overlapping with the HARS group comprising a pancytopenia (Fig. 3). During validation, in particular, miR-574-3p expression values (day 1 after exposure) of the HARS group with pancytopenia still discriminated from the HARS group without pancytopenia (ttest, *p* = 0.009; ROC = 0.96) or the unexposed control (ttest, *p* = 0.003; ROC = 0.94; Fig. 3). Also, HARS group without pancytopenia often revealed gene expression values comparable to H0 (e.g., validation of miR-574-3p, *p* = 0.48; Fig. 3).

## Discussion

We examined the possible clinical diagnostic utility of early radiation-induced gene expression changes on protein-coding mRNA species and noncoding miRNA species in the peripheral blood for the prediction of the late occurring hematological acute radiation syndrome (HARS) comprising a pancytopenia. We aimed to discriminate HARS with pancytopenia from baboons developing a clinically relevant HARS without suffering from pancytopenia. Regarding medical management decision making, it is desirable to know about a developing pancytopenia. During the screening approach, we identified 51 mRNAs and 23 miRNAs. Nine mRNA species and nine miRNA species showed significant differences of HARS groups with and without pancytopenia in comparison to the unexposed controls, but only six miRNA species revealed significant gene expression differences between HARS with pancytopenia and HARS without pancytopenia during the validation step. In particular, miR-574-3p appeared promising and the ROC (0.94) suggested a promising separation of both groups.

The nine mRNAs are coding for proteins involved in cell cycle regulation (*CDCA7L*), modification of the T cell and B cell immune response (*GBP2*, *GLUL*, *HERC5*, *PPP3CC*), erythropoiesis (*GBP2*), and cell migration of cancer and hematopoietic cells (*HERC5*, *HMHA1*). The synopsis of the literature search indicated a general link to hematopoiesis. Three genes were either of unknown function or unrelated. Interestingly, we did not find a reported association to radiation for all the mentioned mRNA species, in opposite to six out of nine identified miRNAs. Unexpectedly, none of the miRNAs were described in the context of hematopoiesis, but miR-146a and miR-378 showed an upregulation after high dose irradiation in vitro (for details, see online resource 2 (a/b)). Five miRNAs (miR-124#, rno-miR-7#, miR-133a, miR-29c, and miR-574-5p) were identified as being involved in radiosensitivity in cancer cell lines/stem cells. Since our miRNA candidates for pancytopenia prediction are not annotated for hematopoiesis but in part to radiosensitivity, it could be hypothesized that alterations in radiosensitivity controlled on a post-transcriptional level might be involved in the development of radiation induced pancytopenia. Noteworthy, since we examined gene expression in the whole blood, we can only speculate about their origin. This could be lymphocytes, granulocytes, and thrombocytes, an interaction of these cells with, e.g., the endothelium or other exposed body areas releasing miRNAs into the blood.

As an alternative to this approach, we already successfully predicted HARS severities defined according to METREPOL [5, 6]. Within these previous studies, we experienced difficulties with the categorization of the four HARS severity scores following the METREPOL protocol [4]. In these previous studies, we simplified the analysis and even merged HARS into new categories, namely developing a HARS (grades 1–3) or developing a more severe HARS (grades 2–3). With this approach, 29 mRNAs could be identified and discrimination was achieved independently with every single of 22 (H1–3) and 7 (H2–3) radiation-induced genes. Clearly, these two HARS categories seem to be more closely correlated to changes in gene expression. Hence, the newly identified candidate genes (in particular, miR-574-3p) for prediction of pancytopenia will add to the already identified gene set for the prediction of different HARS categories and support the medical management decision making.

Some limitations of our study should be kept in mind. We performed gene expression measurements on microarrays and qRT-PCR using human genomic sequences because the baboon transcriptome was not publically available. Given the high homology of both genomes (93%) and previously reports by other groups [11–13], we proceeded as described above. Since we used TaqMan chemistry with human primer and probe sequences (high sensitivity and specificity), it is more likely that we lost some of the detectable human RNA species due to a mismatch with the baboon genome, rather than producing false positives. Also, in preliminary validation studies using human samples from irradiated patients, we could already successfully reproduce most of our selected nine candidate genes from the previous baboon studies implying that differences in genomic sequences represent a minor problem.

The small sample size of our study represents a weakness and surely reduced the number of successfully validated candidate genes. Future work will consider larger sample sizes, and results will be validated on an additional species. As already mentioned, first preliminary validation studies of our previous baboon candidate genes in cancer patients undergoing bone marrow transplantation and TBI regimens are already looking promising and the new gene candidates for prediction of HARS with pancytopenia have to be validated accordingly.

In summary, we identified additional candidate genes (in particular miR-574-3p) for prediction of baboons developing a HARS with pancytopenia. These genes will add to the already identified gene set for the prediction of different HARS categories and, thus, support the medical management decision making.

## Electronic supplementary material

Online resource 1. The figure depicts a flow diagram of included samples, split study design, gene expression measurements and bioinformatics. Numbers in superscript: ^1^samples for stage II: a total of 35 samples for mRNA and another 50 samples for miRNA analysis were used for a validation including samples used in stage I as well. ^2^candidate genes were selected based on the *p*-value, the height and sustained differential gene expression over time. (PPT 102 kb)

Online resource 2 (a/b). Gene description and annotation (Gene ID, gene name and chromosome location) of protein coding mRNAs and miRNAs (from Table 3) are provided in online resource Table 2 (a/b). A literature search (PubMed database/central) examined the association of these mRNA and miRNA species with the hematopoietic system (entries only found for mRNAs) and radiation exposure (entries only found for miRNA species). We employed either NCBI Entrez Gene database (mRNA) or mirBase (miRNA) for gene annotations. The online Resource 2b provides the miRNA ID of the utilized qRT-PCR platform (LDA) and the actual miRNA ID according to the mirBase (mirbase.org, November 2016) which was identified based on the deposited miRNA sequence of the LDA. ^1^miR-720 probably represents a fragment of a tRNA. (XLS 123 kb)
