# Supplementary material for: Gene expression signature for early prediction of late occurring pancytopenia in irradiated baboons
Source: Ann Hematol. 2017 Feb 24;96(5):859–70. doi: 10.1007/s00277-017-2952-7 (PMC5371629; doi:10.1007/s00277-017-2952-7)
Supplement: Supplementary file 1 — The figure depicts a flow diagram of included samples, split study design, gene expression measurements and bioinformatics. Numbers in superscript: 1samples for stage II: a total of 35 samples for mRNA and another 50 samples for miRNA analysis were used for a validation including samples used in stage I as well. 2candidate genes were selected based on the p-value, the height and sustained differential gene expression over time. (PPT 102 kb) [file 277_2017_2952_MOESM1_ESM.ppt]

## Slide 1
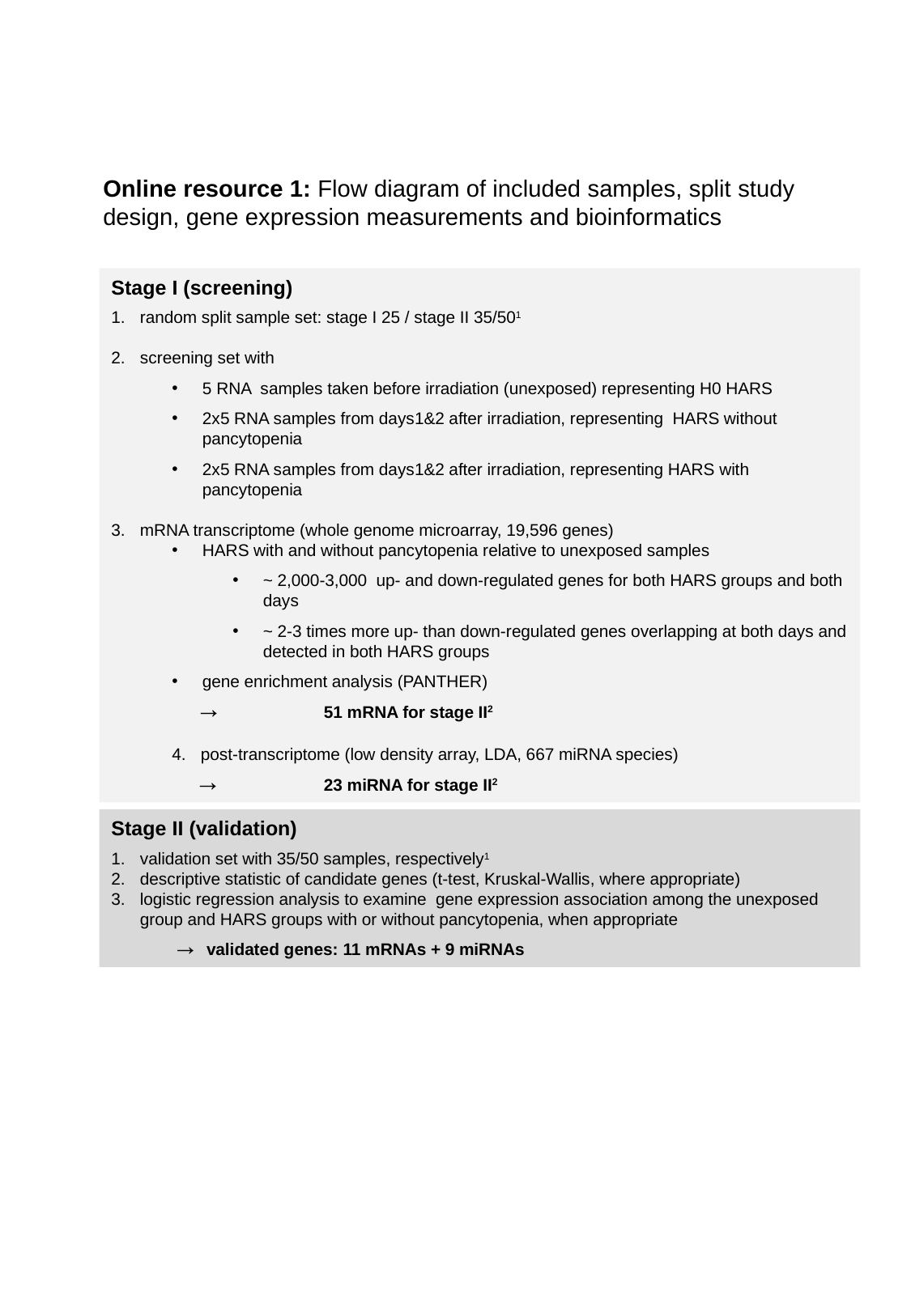

Online resource 1: Flow diagram of included samples, split study design, gene expression measurements and bioinformatics
Stage I (screening)
random split sample set: stage I 25 / stage II 35/501
screening set with
5 RNA samples taken before irradiation (unexposed) representing H0 HARS
2x5 RNA samples from days1&2 after irradiation, representing HARS without pancytopenia
2x5 RNA samples from days1&2 after irradiation, representing HARS with pancytopenia
mRNA transcriptome (whole genome microarray, 19,596 genes)
HARS with and without pancytopenia relative to unexposed samples
~ 2,000-3,000 up- and down-regulated genes for both HARS groups and both days
~ 2-3 times more up- than down-regulated genes overlapping at both days and detected in both HARS groups
gene enrichment analysis (PANTHER)
 →	51 mRNA for stage II2
post-transcriptome (low density array, LDA, 667 miRNA species)
 →	23 miRNA for stage II2
Stage II (validation)
validation set with 35/50 samples, respectively1
descriptive statistic of candidate genes (t-test, Kruskal-Wallis, where appropriate)
logistic regression analysis to examine gene expression association among the unexposed group and HARS groups with or without pancytopenia, when appropriate
→ validated genes: 11 mRNAs + 9 miRNAs
